# Concerns and Challenges Related to Sputnik V Vaccination Against the Novel COVID-19 Infection in the Russian Federation: The Role of Mental Health, and Personal and Social Issues as Targets for Future Psychosocial Interventions

**DOI:** 10.3389/fpsyt.2022.835323

**Published:** 2022-06-14

**Authors:** Anna V. Vasileva, Tatiana A. Karavaeva, Dmitriy S. Radionov, Alexander V. Yakovlev, Igor N. Mitin, Emanuele Caroppo, Sergey I. Barshak, Kirill S. Nazarov

**Affiliations:** ^1^Federal State Budgetary Institution «V. M. Bekhterev National Research Medical Center for Psychiatry and Neurology» of the Russian Federation Ministry of Health, Saint-Petersburg, Russia; ^2^I. I. Mechnikov North-Western Medical State University, Saint Petersburg, Russia; ^3^Federal State Budgetary Institution of Higher Education «Saint-Petersburg State University», Saint-Petersburg, Russia; ^4^Federal State Budgetary Institution of Higher Education «Saint-Petersburg State Pediatric Medical University» of the Ministry Healthcare of Russian Federation, Saint-Petersburg, Russia; ^5^Federal State Budget Institution «National Medical Research Center of Oncology Named After N. N. Petrov» of the Russian Federation Ministry of Health, Saint-Petersburg, Russia; ^6^Federal State Budgetary Military Educational Institution of Higher Education «Military Medical Academy Named After S. M. Kirov »of the Ministry of Defense of the Russian Federation, Saint Petersburg, Russia; ^7^Saint-Petersburg State University of Aerospace Instrumentation, Saint Petersburg, Russia; ^8^Federal State Budgetary Institution “Federal Research and Clinical Center of Sport Medicine and Rehabilitation of Federal Medical Biological Agency”, Moscow, Russia; ^9^Local Health Unit ASL Roma 2, Rome, Italy

**Keywords:** attitudes toward vaccination, COVID-19, coronavirus infection, pandemic, psychosocial interventions targets

## Abstract

**Background:**

Vaccine hesitancy causes serious difficulties in vaccination campaigns in many countries. The study of the population’s attitude toward vaccination and detection of the predictive important individual psychological and social factors defining the vaccination necessity perception will allow elaborating promoting vaccination adherence measures.

**Objectives:**

The aim of this research was to study COVID-19 threat appraisal, fear of COVID-19, trust in COVID-19 information sources, COVID-19 conspiracy beliefs, and the relationship of sociodemographic variables to COVID-19 preventive behavior.

**Methods:**

We carried out a cohort cross-sectional study of the population’s attitude toward vaccination against the novel COVID-19 coronavirus infection, using a specially designed questionnaire for an online survey. Totally, there were 4,977 respondents, ranging in age from 18 to 81 years. Statistical assessment was carried out using the SPSS-11 program.

**Results:**

There were different attitudes toward vaccination. Among respondents, 34.2% considered vaccination to be useful, 31.1% doubted its effectiveness, and 9.9% considered vaccination unnecessary. The survey indicated that 7.4% of respondents were indifferent to the vaccine, while 12.2% deemed it to be dangerous. Nearly one-third (32.3%) of respondents indicated that they did not plan to be vaccinated, while another third (34.0%) would postpone their decision until more comprehensive data on the results and effectiveness of vaccination were available. Only 11.6% of the respondents were vaccinated at the time of the study. Young people were less focused on vaccination compared to middle-aged and elderly people. Receiving information concerning COVID-19 vaccination from healthcare workers and scientific experts was associated with greater vaccination acceptance.

**Conclusion:**

The study results showed that vaccination attitudes interacted with individuals’ mental health and various sociodemographic factors. Insofar as reports of physicians and experts are essential for shaping attitudes to vaccination, the study results inform the selection of target groups in need of particular psychosocial interventions to overcome their vaccine hesitancy.

## Introduction

The COVID-19 pandemic outbreak, which began in early 2020, has become the hitherto most critical event of the century, with a toll of millions of lives. Furthermore, the pandemic has had a serious impact on the mental health and wellbeing of populations around the world (1, 2). State-of-the-art technologies, including mathematical model-based analysis, big-data techniques, and algorithms based on artificial intelligence (AI) have been implemented to cope with this health, economic, and social emergency. In particular, the recent use of AI has significantly accelerated the development of vaccines and treatments. In some circles, this technology has been a source of fear, mistrust, and conspirological beliefs (3). The mathematical model-based analysis enables a better understanding of the factors promoting COVID-19 transmission, supporting a more reliable prediction of the pandemic development: even at its earlier phase, such methods showed that even a moderately effective vaccine would significantly reduce the rate of COVID-19 transmission. The model-based analysis predicted that even a vaccine, such as VES, with greater than 70% efficacy against infection could stop the spread of COVID-19. Conversely, the achievement of herd immunity in the worldwide population would likely have resulted in up to 30 million deaths, while exhausting healthcare resources worldwide (4).

Given the present circumstances of restrictions and risks, rational actors would reasonably be expected to be vaccinated based on their informed appraisal of risk and benefit (5). Nevertheless, we have observed massive disapproval and hostility to vaccination and restriction measures aimed to stop the spread of COVID-19 transmission, culminating in protests in many countries against obligatory vaccination. One of the main expressed concerns is about the safety and possible side effects of the new speedily developed COVID-19 vaccines. Psychological defense mechanisms along with partial reality distortion make mental health issues a serious obstacle in the campaign against the pandemic (6, 7). The spread of COVID-19 infection is accompanied by a massive infodemic, with misinformation spreading much faster than the virus itself and having a great effect on public acceptance of vaccination another other public health measures (8–10).

In particular, the involvement of the new technologies aimed to stop the pandemic is dramatically augmenting public mistrust, conspirological theories, and vaccine hesitancy as detected by digital media portals (9, 11–14). Vaccine hesitancy is a matter of great concern to the World Health Organization (WHO). Even in 2015, the WHO, 2015 Strategic Advisory Group of Experts on Immunization identified vaccine hesitancy as a delay in acceptance or refusal of vaccination, despite the availability of vaccination services. Vaccine hesitancy can differ in intensity and involves various conspirological beliefs, such as the contention that it serves as a tool of mass chipping and pervasive social control. The spread of misinformation only increases vaccine hesitancy, and WHO announced this in 2019 (thus, prior to the pandemic) to be one of ten main global health threats and a massive obstacle to achieving population immunity against disease (15, 16). In the Russian Federation, the Moscow-based Gamaleya Research Institute of Epidemiology and Microbiology applied its experience in platform research for Ebola and Middle East respiratory syndrome vaccines toward the development of Gam-COVID-Vac (Sputnik V), a heterologous rAd26 and rAd5 vector-based COVID-19 vaccine. This initially demonstrated a good safety profile and induced strong humoral and cellular immune responses in participants in phase 1/2 clinical trials. The interim analysis of the phase 3 trial of Gam-COVID-Vac showed 91.6% efficacy against COVID-19 and good tolerance (17, 18). Experience has shown that because of vaccine hesitancy and mythological thinking, vaccine availability does not ensure mass population vaccination.

The WHO recommends that each country study its climate of vaccine hesitancy and develop targeted strategies, including brief psychosocial interventions or campaigns, to increase vaccination acceptance (19). Our first study, conducted during the early months of Sputnik V vaccination, preceding the public educational campaigns, showed that only 12.2% of respondents had been vaccinated and more than 60% had some degree of hesitancy. Recent studies have shown the importance of receiving information about COVID-19 vaccination from healthcare workers for vaccination acceptance as well as the perceived severity of COVID-19 (20). The other research emphasized the impact of COVID-19 threat appraisal on the COVID-19 preventive behavior adherence (5). As mentioned above, the COVID-19 experience is an important factor in the study of attitudes toward vaccination. Understanding the factors that determine vaccine hesitancy is essential for the planning of brief, targeted psychosocial interventions (21). Understanding the sources of unwillingness to be vaccinated is crucial for elaboration of appropriate measures to improve vaccination adherence.

### Objectives

The objectives of this study are identification of the predictive significant individual psychological and social combination of variables, determination of vaccination attitude at the beginning of the vaccination campaign in the Russian Federation, and elaboration of the model that can predict vaccination attitude.

### Hypothesis

Different vaccination attitudes are connected with specific respondents’ characteristics such as sociodemographic factors, gender, social and educational status, personal COVID-19 experience, presence of anxiety and worries, wellbeing status, personal beliefs about vaccination usefulness or harm, and attitude to one’s health. The identification of these variables’ patterns allows the prediction of vaccination attitudes in different population groups for the further development of the targeted public health programs aimed to increase vaccination acceptance.

## Materials and Methods

A cohort cross-sectional study of the population’s attitude toward vaccination against the COVID-19 coronavirus was carried out using a specially designed questionnaire for a mass online survey. The sample was collected through study promotion *via* the most popular social media (VK, WhatsApp, Viber, Facebook, and Telegram). Considering the importance of opinions of healthcare professionals, we targeted our recruitment toward medical professional portals and mailing lists. In addition, to obtain a group of respondents with preexisting mental health conditions, we promoted the study through mailing list databases and *via* a partnership with the Russian Society of Psychiatrists and patient organizations. The total sample of 4,172 respondents included 42.2% with higher medical education and 20.5% with a previous history of mental disorders, attested by their presence on mailing lists. The study was attended by respondents from 64 of the 85 districts of the Russian Federation. Most cities with a population of 1 million or more were represented, namely, St. Petersburg, Moscow, Novosibirsk, Chelyabinsk, Kazan, Ufa, Rostov-on-Don, Voronezh, and Krasnodar (refer to Table 1). Approximately 40% of respondents lived in smaller settlements (less than 500,000 people) but were nonetheless able to participate given the broad Internet penetration. The survey was extended from 5 March to 5 June 2021.

**TABLE 1:** Sociodemographic characteristics of the study group.

Settlement	Sample (n)	Percentage (%)
In the countryside	324	7.8
In a city with a population of less than 100,000 people	478	11.5
In a city with a population of 100,000 – 500,000 people	931	22.3
In a city with a population of 500,000 -1,000,000 people	844	20.2
In a city with a population of more than 1,000,000 people	1,595	38.2
Total sample	4,172	100

The questionnaire allowed us to obtain sociodemographic, anamnesis, clinical data, and psychological characteristics of respondents while assuring anonymity. The complete questionnaire was divided into the following sections:

Section 1 included sociodemographic parameters such as age, sex, education, social status, the population of the place of residence, type of activity, family, and a financial statement.

Section 2 included attitude toward vaccination against the novel coronavirus infection, the incidence of previous novel coronavirus infection among respondents and their immediate family/social circle, the general attitude toward vaccination and specifical vaccination against the novel coronavirus infection, if the respondent was vaccinated, and whether he/she plans to be vaccinated, willingness to recommend that relatives and friends be vaccinated (which greatly affects the broader formation of attitudes to vaccination), the presence of anxiety associated with the risk of getting sick and with the risk of possible complications from vaccination, and the presence of somatic and mental disorders that might affect the attitude to vaccination.

Section 3 was comprised as follows:

1. A questionnaire containing beliefs about vaccines and vaccination. The Vaccination Attitudes Examination (VAX) Scale, the double translation of the questionnaire, has been made before its implementation in the study (22).
2. The General Health Questionnaire, GHQ12, evaluating an individual’s psychological wellbeing and distress D. P. Goldberg (1972). The adaptation of the Russian version was made by Burlachuk L. F. in 2005 (23, 24).
3. Health Attitude Questionnaire (R. A. Berezovskaya, 2005).

Participation in the study was anonymous and voluntary. The Independent Ethical Committee at the V. M. Bekhterev National Medical Research Center approved the study for Psychiatry and Neurology (EK-I-31/21 from 25 February 2021). Before filling out the questionnaire, the respondent had the opportunity to get acquainted with the goals and conditions of the study and to give informed consent to participate by marking in the appropriate paragraph. After filling out the questionnaire, the respondent could send the completed data, or withdraw from the survey without the inclusion of their responses in the survey. Only surveys with 100% completion were analyzed. Analysis and assessment of the survey’s results were carried out within 2 months after the launch of Russia’s mass vaccination campaign.

The inclusion criteria were as follows:

1. Over the age of 18
2. Informed consent to participate in the study
3. Ability to read Russian and fill out an online questionnaire

The ex/non-inclusion criteria were as follows:

1. Age less than 18 years
2. Inability to understand the text and content of the questionnaire

The exclusion criteria were as follows:

1. Participants declining at any stage to participate in the survey

Statistical assessment was carried out using the SPSS-11 program. Descriptive data analysis and two-dimensional (cross-tabulation) statistical analysis were used. Statistical confidence was judged according to the F-test (Fisher’s criterion; φ). The procedure for data collection excluded the possibility of duplication. The significance level was defined as l φ = 0.05. Results from 4,977 people aged 18–81 years were included, with a mean (SD) age of 37.58 (13.56) years. Of the population, 1,393 (28.0%) were men and 3,584 (72.0%) were women. The study included all age groups of the adult population, according to the WHO classification: young aged (18–44 years)—3,445 (69.2%); middle-aged (45–59 years)—1,178 (23.7%); elderly aged (60–74 years)—343 (6.9%); and extremely old aged—11 (0.2%). The elderly and extremely elderly groups were combined to yield 354 respondents (7.1%). The educational attainment of respondents was 23 (0.5%) with secondary education, 987 (19.8%) with further education, 387 (7.8%) with incomplete higher education, 2,603 (52.3%) with higher education, and 977 (19.6%) with two higher educations or academic degrees.

### Social Status of Respondents

Among the 4,977 respondents, 921 (18.5%) were students, 3,426 (68.8%) were working, 249 (5.0%) were business owners, and 153 (3.1%) were homemakers. There were 160 (3.2%) pensioners, 57 (1.1%) unemployed, and 11 (0.2%) living on benefits. Since the presence of technical knowledge is important for the formation of attitudes toward vaccination, medical education and medical specialty were separately considered. The total sample included 2,153 (X%) health workers, among which 908 people (42.2%) were physicians, 291 (13.5%) nurses, 59 (2.7%) paramedics, 28 (1.3%) medical attendants, 498 (23.1%) medical students, 122 (5.7%) administrative staff, and 247 (11.5%) other health workers. Among the respondents, 859 (20.5%) suffered from anxiety disorders, of which 411 (9.9%) had suffered from depression and 126 (3.0%) mainly had psychotic mental disorders.

## Results

### COVID-19 Personal Experience

About half (*n* = 2,909; 58.4%) of the respondents did not suffer from a novel coronavirus infection since COVID-19 outbreak, asymptomatic infection (*n* = 390; 7.8%), mild illness (*n* = 910; 18.3%), moderate illness (*n* = 670; 13.5%), and severe illness (*n* = 98; 2.0%). Restrictive measures introduced for the older population and the very old proved to be effective; among these age groups, a significantly higher proportion of patients did not experience infection with the virus (67.8%), compared to rates in the young (58.7%) and middle-aged (54.8%) subgroups. Reliable differences are observed both between the young and the elderly (*p* < 0.01; φ = 2.798) and between the middle aged and the elderly (*p* < 0.01; φ = 3.572).

It was also assessed whether close contacts of individuals had suffered from a novel coronavirus infection as well as the severity and course of the disease. Respondents were allowed to answer the question in a multiple-choice format. One-third of respondents’ relatives (*n* = 1,654; 33.2%) suffered asymptomatic infection; 3,584 (72.0%) experienced mild illness. Almost half of respondents’ relatives (*n* = 2,123; 42.7%) suffered from severe illness (hospitalization was required) and a large number of relatives (*n* = 1,015; 20.4%) died as a result of coronavirus infection. Only 647 (13.0%) of relatives did not have this infection.

### COVID-19 Vaccination Attitude

Among the population, there were different views and ideas about the benefits and need for vaccinations in general and vaccination against various infections. Responses were distributed approximately evenly across four types of vaccination attitudes. No significant differences by age group were found for this variable.

Among respondents, 1,309 (26.3%) people tried to avoid any vaccination, 1,370 (27.5%) were vaccinated sometimes, 855 (17.2%) were always observed, and 1,443 (29.0%) were vaccinated at the recommendation of specialists. The main objective was to assess the attitude of the population to vaccination against the novel coronavirus infection. A third of those respondents (*n* = 1,703; 34.2%) considered vaccination useful, while a third (*n* = 1,550; 31.1%) doubted its effectiveness, 9.9% (*n* = 492) of respondents considered vaccination unnecessary, and 12.2% (*n* = 609) considered it to be dangerous. Indifferent attitude toward vaccination was formed in 7.4% (*n* = 367) of respondents. Some other opinions were held by 5.1% (*n* = 256). There is a relationship between the attitude to vaccination and the age of the respondents (refer to Table 2). Old and very old respondents considered vaccination to be unnecessary, dangerous, or doubtful in its effectiveness less often than young or middle-aged respondents (*p* < 0.01).

**TABLE 2:** COVID-19 vaccination attitudes among different age groups.

The attitude of the population to vaccination against COVID-19		Age groups (WHO)			Total sample n (%)
		Age group I (ages from 18 to 44)	Age group II (ages from 46 to 54)	Age group III (ages from 60 to 89)	
Vaccination	is	411	71	10	492 (9.9%)
unnecessary		11.9%	6.0%	2.8%	
		I and II. *p* < 0.01. φ = 6.192 I and III *p* < 0.01. φ = 6.593			
Vaccination	is	957	542	204	1703(34.2%)
useful		27.8%	46.0%	7.6%	
		I and II. *p* < 0.01. φ = 11.259 I and III. *p* < 0.01. φ = 10.965 II and III. *p* < 0.01. φ = 3.828			
Vaccination	is	474	116	19	609 (12.2%)
dangerous		13.8%	9.8%	5.4%	
		I and II. *p* < 0.01. φ = 3.674 I and III. *p* < 0.01. φ = 5.232 II and III. *p* < 0.01. φ = 2.772			
Doubts about	the	1113	343	94	1550 (31.1%)
effectiveness		32.3%	29.1%	26.6%	
		I and II. *p* < 0.05. φ = 2.044			
		I and III. *p* < 0.05. φ = 2.224			
Indifferent attitude		325	32	10	367 (7.4%)
		9.4%	2.7%	2.8%	
		I and II. *p* < 0.01. φ = 8.681 I and III. *p* < 0.01. φ = 5.142			
Others		165	74	17	256 (5.1%)
		4.8%	6.3%	4.8%	
Total sample n (%)		3445	1178	354	4977 (100%)
		100.0%	100.0%	100.0%	

**FIGURE 1:**
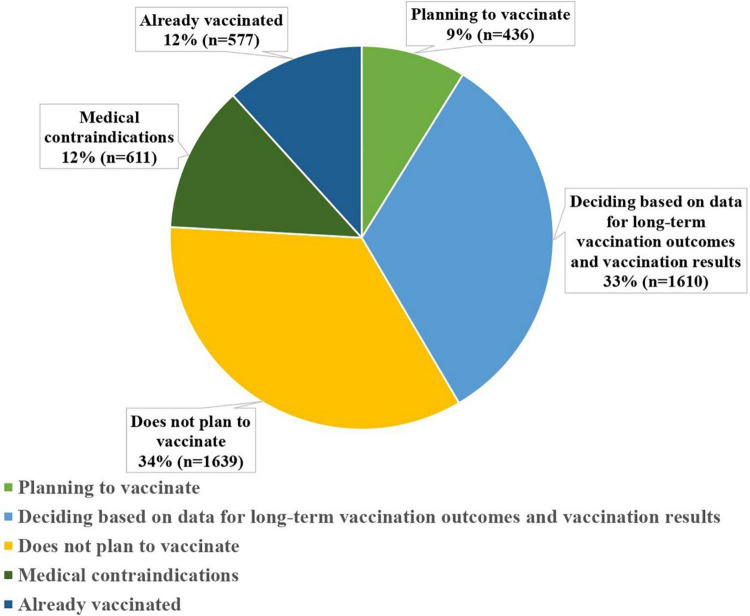
Vaccination behavior types as presented in the study group.

There are also sex differences in vaccination attitude; more men than women consider vaccination to be useful (*p* < 0.01; φ = 6.461), and there are fewer respondents among men who doubt the effectiveness (*p* < 0.01; φ = 5.923). Among women, there is a greater percentage of those who consider vaccination to be dangerous (*p* < 0.01; φ = 3.389). Most of the respondents do not have fears related to possible vaccine shortages (*n* = 3,579; 85.8%). Such concern was noted by 500 people (12.0%), with 93 (2.2%) respondents having very significant concerns about vaccine shortages. When the questionnaire asked regarding specific actions of respondents in attitude to their own vaccination, 577 (11.6%) respondents noted they have already been vaccinated, 661 (13.3%) planned to vaccinate shortly, 1,693 (34.0%) are going to make decisions based on data on long-term outcomes and vaccination results, 1,610 (32.3%) indicated they do not plan to vaccinate, and 436 (8.8%) have medical contraindications (refer to Figure 1).

There are significant differences in the age group. Among young people, there are more respondents who do not plan to be vaccinated than among middle-aged people (*p* < 0.01; φ = 11.288) and the elderly (*p* < 0.01; φ = 10.499), less who plan to be vaccinated in the near future (*p* < 0.01; φ = 4.978; φ = 5.679), and less already vaccinated (*p* < 0.01; φ = 7.526; φ = 4.264). The proportion of respondents who would recommend vaccinations to friends and relatives and their relation to attitude to this preventive measure is important for assessing respondents’ attitudes to vaccination against the novel coronavirus infection. Less than a third of respondents (1,340; 26.9%) noted that they would recommend a vaccine; 1,986 (39.9%) respondents are not ready to recommend it, and 293 (5.9%) intend to actively dissuade others, and 1,358 (27.3%) have not yet decided. Respondents who are ready to recommend vaccinations to friends and relatives consider it useful for the most part (88.1%). Respondents who replied that they will try to dissuade relatives or did not plan to recommend it consider it dangerous (54.3% and 19.4%, respectively), unnecessary (25.3% and 16.1%), or ineffective (16.0% and 43.2%) (refer to Supplementary Table 1).

The impact of sociopsychological factors on the attitude toward vaccination.

The attitude toward vaccination is manifested and largely formed depending on the results shown by vaccination in different countries, i.e., on data provided by the media, official state, and medical sources. It was noted assessing the respondents number who were interested in the course of vaccination, monitor the results and effectiveness of vaccinations, 723 (14.5%) people closely follow, they report that they monitor to some extent, a third of respondents (*n* = 1,478; 29.7%); 1,255 (25.2%) are somewhat less interested. A third of respondents (*n* = 1,521; 30.6%) do not monitor the results of vaccination. Those respondents who consider vaccination unnecessary (63.0%) and are indifferent (56.4%) are more interested in vaccination results. A significant number of respondents who consider the vaccine dangerous or doubt its effectiveness continue to be interested in the results (58.5% and 67.0%, respectively).

The likely cautious population’s attitude toward vaccination may be due to fear of perceived complications. Only 946 (19.0%) people are not afraid of possible complications, 1,342 (27.0%) are slightly feared, 1,163 (23.4%) are moderately feared, 801 (16.1%) are greatly feared, and 725 (14.6%) are very much feared. In the group of respondents who are very afraid of complications from vaccination, the greater proportion of those generally consider it dangerous (40.6%) or doubt its effectiveness (30.3%) (refer to Supplementary Table 2). Notably, doctors are reliably less afraid of complications from vaccination than all other categories of medical workers and respondents who do not work in the medical field (*p* < 0.01).

Of the total sample, 1,485 (29.8%) people suffer from any chronic disease (e.g., hypertension, diabetes mellitus, bronchial asthma, obesity, or being overweight). Among them, some people are very afraid of complications (*p* < 0.01; φ = 25.621). In addition, significantly less than those who consider vaccination unnecessary-87 – 5.9%) (*p* < 0.01; F = 6.585) than necessary.

The study demonstrated that vaccination attitude is influenced by individuals’ mental health. By filling out the questionnaire, respondents were able to indicate the presence of a known mental health disorder based on a previously given diagnosis. Individuals with anxiety (*p* < 0,01; φ = 6.584) and depressive disorders (*p* < 0.01; φ = 4.671) had significantly more concerns about possible vaccination complications than healthy respondents. In contrast, people with anxiety disorders more than others evaluated immunization as a useful measure against COVID-19 (*p* < 0.01; φ = 6.352); among the depressive patients, more respondents had doubts about vaccination efficacy than in the other groups (*p* < 0.01; φ = 5.149). Patients with other mostly psychotic mental disorders to some degree were more indifferent to vaccination (*p* < 0.01; φ = 7.437). In addition, there are significantly fewer people who consider vaccination unnecessary – 87 (5.9%) (p < 0.01; φ = 6.585). In all groups of people who fear for the health of relatives, the proportion of those who consider vaccination useful is significantly higher compared to those who do not have such fears (*p* < 0.01; φ = 7.263; φ = 11.451; φ = 10.76; φ = 8.56). There is a relationship between the vaccination attitudes and the fear of the severity of contracting coronavirus infection (refer to Supplementary Table 3).

Among those who are not afraid of contracting coronavirus, the percentage of those who consider vaccination unnecessary is **higher** compared to those who are afraid of getting sick (*p* < 0.01 compared to all groups). Almost half of those who fear getting sick moderately (45.0%; *p* < 0.01; φ = 12.328), strongly (52.6%; *p* < 0.01; φ = 6.15), and very strongly (47.7%; *p* < 0.01; φ = 5.338) are confident in the utility of vaccination, reliably more than those who are not afraid to get sick. These groups of respondents have less doubt about the effectiveness of the vaccine. A slightly more than a third of respondents (*n* = 2,000; 40.2%) noted that they do not experience anxiety at all due to the current situation with coronavirus, rarely experience anxiety (*n* = 1,247; 25.1%), sometimes (*n* = 1,326; 26.6%), often (*n* = 302; 6.1%), and very often (*n* = 102; 2.0%). Among those who often and very often experience anxiety due to the situation with coronavirus, a large number of those who consider the vaccine useful (44.7% and 52.9%; respectively) are significantly more than those who do not experience anxiety (25.4%) (*p* < 0.01; φ = 6.625; φ = 5.645). Individuals who very often experience anxiety are less doubtful of vaccine efficacy, at only 22.5%, which is lower than in other groups where anxiety was less common (*p* < 0.05; φ = 2.476). The fear of dying due to coronavirus is not experienced by 2,552 (51.3%) respondents, is experienced less by 1,871 (37.6%) respondents, is experienced strongly by 345 (6.9%) respondents, and is experienced very strongly by 209 (4.2%) respondents. The preferred information sources defining the vaccination attitudes and behavior in the population were studied (refer to Table 3). The respondents were provided with a list of the main information sources with multiple-choice options. The majority of the respondents preferred reports from scientists, physicians, and other experts (81.2%). Opinions of family members and friends (22.9%), the media (20.9%), and social networks (16.3%) have a significantly lower influence. Statements and opinions of public figures have the lowest level of public confidence (10.6%), significantly lower compared to scientists and physicians (p < 0.01; φ = 78.918).

**TABLE 3:** Information sources influencing the formation of attitudes toward vaccination.

Opinion about coronavirus infection and vaccination is determined by:		Sample (n)	Sample (%)
1	Reports by scientists, physicians and other experts	4,042	81.2
2	Opinion of famous people and public figures	527	10.6
	1 and 2 *p* < 0.01; φ = 78.918		
3	Media	1,041	20.9
4	Opinions of my family members and friends	1,140	22.9
5	Information in social networks	809	16.3

There is a relationship between the education of respondents and the proportion of people who noted the significant influence of a particular information sources. Among people with higher education, a significantly larger number noted the significant influence of scientists, doctors, and experts on their relationship to vaccination compared to those who had further education (85.0% and 64.7%, respectively, *p* < 0.01, φ = 12.76). Among those with further education, respondents noted the influence of the media (31.4%) and social networks (24.0%) are more than among those with higher education (18.4%: *p* < 0.01; φ = 8.106 and 14.2%: *p* < 0.01; φ = 6.715, respectively).

### Vaccination Beliefs

A questionnaire on attitudes to vaccination was included as a separate block of the questionnaire, with 12 questions and four scales, namely, “Distrust of the benefits of the vaccine,” “Distrust of unforeseen consequences in the future related to the vaccine,” “Concerns about commercial speculation,” and “Preference for natural immunity.” Respondents noted their attitude to the statements in the questionnaire on a six-point scale ranging from 1 (absolutely disagree) to 6 (absolutely agree). The average value was calculated. Indicators ranging from 1 to 3 indicate disagreement with the statements, indicators ranging from 3 to 4 indicate neutrality, and indicators ranging from 4 to 6 indicate consent with the statements. Responses from respondents in different age groups were also studied. Those surveyed by all age groups believe the vaccine has not been sufficiently studied and can negatively affect health. Among respondents, there is no support for the idea of commercial speculation on vaccination that vaccines are more beneficial to pharmaceutical companies than the population, and the vaccination program itself is profane (2.96). Young people do not believe that vaccination gives them a sense of safety (2.95), unlike middle-aged and elderly people who agree that vaccines can stop serious infectious diseases (4.03 and 4.29).

### Discriminant Analysis Results

To build a model, the respondents’ answer about their attitude toward vaccination (variable Q2_015) has been chosen as a group variable. According to the values of this variable, the observations were divided into 6 groups of respondents (refer to Table 4).

**TABLE 4:** Respondents’ groups as divided by the factor of their attitudes toward vaccination.

Q2_015 = 1	Respondents who consider vaccination unnecessary (group 1)
Q2_015 = 2	Respondents who consider vaccination useful (group 2)
Q2_015 = 3	Respondents who consider vaccination dangerous (group 3)
Q2_015 = 4	Respondents who doubt vaccination effectiveness (group 4)
Q2_015 = 5	Respondents who are indifferent toward vaccination (group 5)
Q2_015 = 6	Respondents who have others attitude toward vaccination (group 6)

The analysis of the questionnaire results, based on the descriptive statistic methods and contingency tables revealed that the respondents’ attitudes toward vaccination, can be influenced by a number of variables. These variables, conditionally combined into 6 semantic groups, are shown in Supplementary Table 4.

Notably, a number of variables presented in Supplementary Table 4 were calculated based on the respondents’ answers. These are the variables Q1_009a from the first group of variables and Q2_014a from the second group of variables. The variables included in the 4th to 6th groups of variables are the values of the scales of the previously mentioned questionnaires VAX, GHQ-12, and attitude to one’s health questionnaire (R. A. Berezovskaya) and also were calculated based on the respondents’ answers.

The task was to develop a mathematical-statistical model that could classify the respondent into one of the 6 groups presented in Table 4 based on the values of the variables presented in Table 4.

To build the model, the initial data set of 4,977 observations was analyzed, and 83 observations containing incomplete data were excluded. The remaining 4,894 observations were divided into two parts, namely, the training sample (*N* = 2447) and control sample (*N* = 2447).

To develop the model, the observations from the training sample were used. To check the quality of the developed model, observations from the control sample were used.

Discriminant analysis was used to build this model. The first five canonical discriminant functions were used in the calculated model. Thus, the first discriminant function provides 88.7% of the prognosis and the second for 7.5%. The sum of the first two discriminant functions provides a 96.2% prognosis (refer to Table 5).

**TABLE 5:** Classification model confusion matrix (% of true and false classification results in the control sample data).

Discriminant function	Eigen value	% of variance	Cumulative%	Canonical correlation
1	1,010a	88,7	88,7	,709
2	,086a	7,5	96,2	,281
3	,024a	2,1	98,3	,154
4	,014a	1,2	99,5	,117
5	,005a	,5	100,0	,072

Supplementary Table 5 shows the unstandardized coefficients of the canonical discriminant functions for each of the variables used in the model. Group means of non-standardized canonical discriminant functions (group centroids) for the groups of respondents described in Table 4 are presented in Supplementary Table 6.

Supplementary Table 5 allows you to calculate the values of discriminant functions 1–5 based on the variables presented in Supplementary Table 4. The obtained values of discriminant functions 1–5 are compared with group centroids (in Supplementary Table 6). Thus, the respondents are classified, i.e., assigning it to one of the six groups under consideration.

**FIGURE 2:**
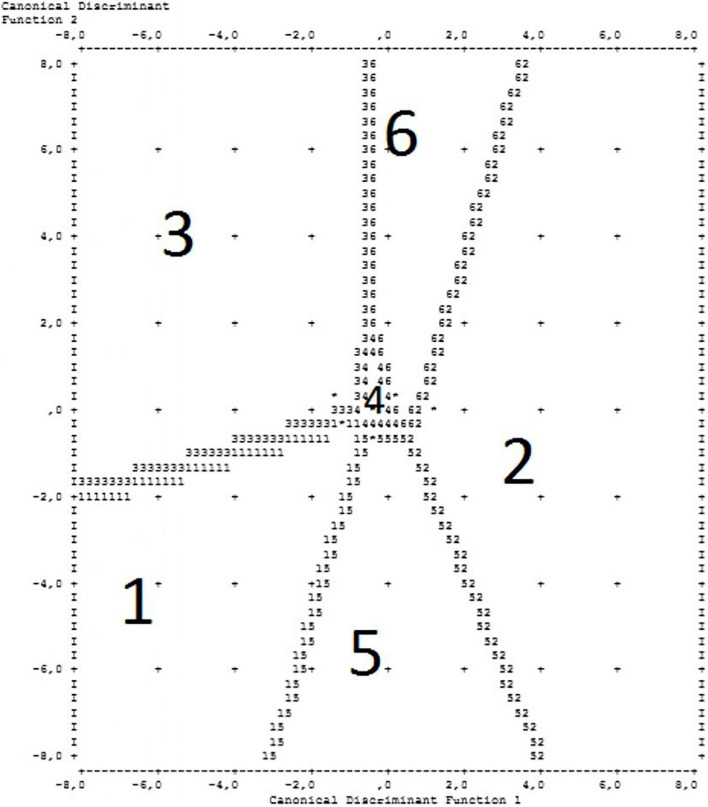
Groups and groups’ centroid location on the discriminant functions axis.

**FIGURE 3:**
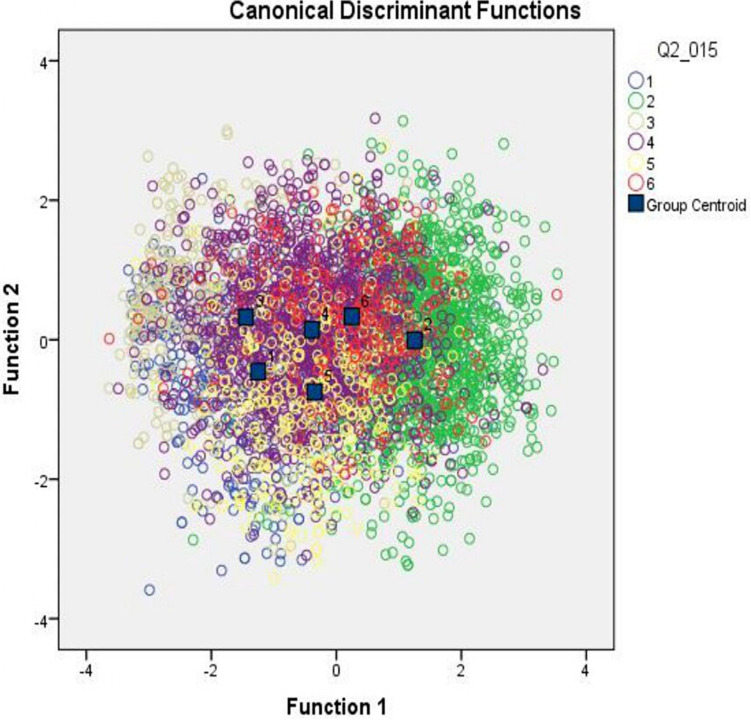
Groups and groups’ centroid location on the discriminant functions axis.

Figures 2, 3 present the groups and groups’ centroid location on the discriminant functions axis. Consequently, group 1 (respondents consider vaccination to be unnecessary), group 5 (respondents who are indifferent toward COVID-19 vaccination), and group 2 (respondents who think vaccination to be useful) were located on the first discriminant function axis. At the same time, group 1 follows group 3 (respondents who consider vaccination to be dangerous) on the second discriminant function axis.

Thus, a set of discriminant functions was developed that allows the recognition (classification) of the respondent’s attitude toward vaccination based on the analysis of his/her answers to a number of questions from the proposed questionnaire.

To evaluate the effectiveness of the developed model, the data in the control sample were classified using the developed discriminant functions. Supplementary Table 7 presents the results of the quality assessment of the developed classification model. These results are presented in the form of a matrix containing the percentage of correct and incorrect classifications of control sample data. Computations showed that 45.7% of the primary groups were classified correctly.

It is worth mentioning that the model has a high percentage of correct classification (69.9%) for group 2 (respondents who thought vaccination to be useful), and a relatively high percentage of the correct classification (50.5%) for group 3 (those who consider vaccination dangerous). At the same time, the model hardly differentiates groups 1 and 3. However, if we classify this group as a vaccination non-compliant population, the percentage of correct allocation can be acceptable.

## Discussion

During the start-up phase of prevention programs against the novel coronavirus infection, participants were surveyed about their views on vaccination. A third of respondents consider vaccination useful, while the same portion doubts its effectiveness. About a quarter of respondents perceive it as unnecessary, dangerous, or indifferent. These perceptions influence behavior and decision-making regarding one’s own vaccination. A third of the entire sample notes that they do not plan to vaccinate, another third doubts the decision and focuses on the more distant results of the vaccination program conducted in the country, 11.6% are already vaccinated, and 13.3% plan to vaccinate shortly. The percentage of Russian citizens who were unwilling to get a COVID-19 vaccine was similar to the results from a European survey published in 2020 of adults across seven European countries (19). Our results suggest more positive vaccination attitudes among older adults (65 years and older) and middle-aged adults compared to young people. The COVID-19 vaccine-related attitudes research in Canada has shown similar results of some degree of vaccine hesitancy in 60% of the respondents, with a significant association with younger age (18–39 years). In a similar United Kingdom study, the uncertain group made up nearly a quarter, with a large proportion of younger age respondents constituting the 14% who were unwilling to get vaccinated (6.21). The study results showed that men considered the vaccine useful more often and had a lower proportion of those with vaccine hesitancy compared to women. Women had negative attitudes toward COVID-19 vaccination in a large number of studies conducted worldwide, which can most likely be attributed to beliefs that the vaccine can have a negative impact on reproductive function (23–27). Lack of trust in the vaccine’s benefits and efficacy as well as concerns about the novelty, safety, and unknown side effects comprise the key obstacles to vaccine willingness.

Overall, respondents’ concerns are mostly related to fear of possible negative complications from the vaccine, which are currently unobvious or unknown (4.17). This echoes results obtained in numerous other studies; newness, safety, and potential side effects can be considered universal concerns, making an impact on achieving COVID-19 public immunity (14, 28, 29). The absence of COVID-19 contamination concerns, poor compliance with epidemiological guidelines, and low knowledge about COVID-19 and possible complications is associated with lower vaccination adherence. The same tendencies were found in a number of other studies (30, 31). The attitude toward vaccination determines the population’s activity and intention to recommend vaccination to their loved ones and friends. Only less than a third of those interviewed are willing to do the latter. Most respondents will experience, to varying degrees, fear of getting a coronavirus infection, concern for the health of their relatives, and anxiety due to the current situation with coronavirus in general. The presence of these experiences contributes to a more positive attitude toward vaccination. Considering the higher mortality rate and the difficulties in compliance with protective behavior due to cognitive defects, there is an urgent need to develop personalized psychosocial interventions to improve vaccination adherence in mentally ill patients (32–34). Among the factors influencing vaccination attitudes, the reports of scientists, physicians, and experts in the field are of greater importance, which generally reflects the public’s confidence in the information obtained from these sources. In general, among the population, the level of confidence in the vaccine can currently be estimated as average. Among young people, the idea of the benefits of the vaccine is viewed with more skepticism than among middle-aged and elderly people. Most of the concerns relate to possible negative unforeseen consequences of vaccination that may result in the future. The analysis showed an association of certain sociodemographic characteristics and individual experiences of the COVID-19 pandemic with attitudes toward vaccination.

The implementation of discriminant analysis in the large sample analysis allowed us to make a mathematical model. It can be used to predict an individual’s attitude toward vaccination against the novel coronavirus infection based on the connected variables group. The use of predictive models can determine specific population groups and implement public health programs aimed to increase vaccination adherence at the early stages of vaccination campaigns. Considering the factors that separate the groups provides the opportunity to elaborate on targeted public health strategies and correct their content. As an example, people having concerns about possible vaccination side effects should be provided with information about vaccination consequences, and people with indifferent vaccination attitudes should be addressed with motivation enhancing interventions.

## Conclusion

The study results show the population’s vaccination attitude in the first 2 months after its start. The data analysis revealed the impact of specific social demographic characteristics, personal COVID-19 pandemic experience, and mental health status on the vaccination attitude rate.

1) At the beginning of the vaccination campaign, 32.4% of the respondents considered it useful; 31.1% doubted its effectiveness; 9.9% considered vaccination unnecessary; 12.2% deemed it dangerous; and 7.4% are indifferent toward vaccination.

2) Higher vaccination adherence is associated with elderly and senile age, negative COVID-19 personal experience (respondents themselves or their close ones had severe COVID-19 cases, or died), somatic diseases, anxiety disorders, and healthcare worker professions.

3) Vaccine hesitancy is mainly determined by fear of possible adverse side effects and distrust of the benefits of vaccination.

4) The mathematical model can statistically accurately classify patients in one of the defined groups, using analysis of the following variables: gender and social characteristics, COVID-19-associated personal experience, presence of somatic diseases and mental health problems, COVID-19-associated anxiety, presence/absence of the specific general vaccination beliefs, psychological wellbeing and distress level, and attitude to one’s health.

Given the importance of creating accurate perceptions among the population concerning the fight against the new coronavirus infection, psychosocial interventions aimed at increasing adherence to vaccination should address targets that are associated with a wary attitude of the population toward preventive measures. Considering the relatively large proportion of uncertain individuals in the sample, future research should investigate the factors defining the uncertainty about vaccination to build the most promising target for psychosocial interventions aimed to improve immunization. Concerns about vaccine safety and novelty, identified in the study as important factors in vaccine hesitancy, should be included as the main targets in the tailored public health vaccination campaign. Simple, clear explanations of how the new technologies can speed up vaccine creation and a balanced discussion of immunization risks and benefits should be emphasized. Given that healthcare professionals and scientists are more trusted sources, these key opinion leaders should be more involved in the vaccination campaign. The additional refreshment professional training for healthcare workers focused on infectious diseases and immunology can significantly improve their own vaccine hesitancy and make them knowledgeable and encouraging in their dialog for vaccine uncertain and unwilling populations. For specific social groups that are associated with vaccine hesitancy, including younger people and women, the public health messaging should be tailored accordingly to provide transparent and clear-cut information about vaccination safety and address the female fears about possible infertility and vaccination teratogenic effects. For the young population, relevant celebrities should be involved in the vaccine campaign, and the negative social consequences of the prolonged pandemic should be emphasized to empower the youth that their decisions and behavior matter in the fight against the COVID-19 pandemic.

Taking into account the higher mortality rate and difficulties in compliance with protective behavior due to cognitive deficits, there is an urgent need to develop motivating psychosocial interventions to improve vaccination adherence in mentally ill patients (12). It could be recommended to organize the COVID-19 vaccination centers in the framework of mental health services to provide timely immunization to patients suffering from psychotic disorders. When researching vaccination attitudes, it is vital to involve population groups with more nuanced decision-making processes and vaccination unwillingness and uncertainty understanding in order to design psychosocial interventions accordingly.

### Study Limitations

The major limitation of this cross-sectional study is that it represents one snapshot in time. The responses were collected at the beginning of the mass vaccination campaign and before any announcements about the success and safety of mass COVID-19 vaccination could be made.

The survey recruited participants from social media platforms and through mailing lists. There could be a component of selection bias as participants volunteered to participate in the research surveys through an electronic platform, which may lead to an increased selection of individuals with higher involvement in the COVID-19 pandemic, resulting in an underestimation of vaccine hesitancy. The availability of “Sputnik V” in all regions of the Russian Federation should inspire the government to encourage the population to get vaccinated, which can differ from other countries.

Our research also has some limitations regarding instruments. Since data collection took place over the Internet, the population study design does not permit the usage of psychometric instruments to evaluate anxiety symptoms’ intensity and their interrelationship with attitudes toward vaccination. Further research in smaller groups that include patients with anxiety and other mental disorders should be designed with the use of appropriate psychometric scales to obtain more specific information about psychopathological disturbances. The “attitude to one’s health” questionnaire used in this study is an original Russian instrument that cannot be compared to the results of similar international studies. It can be useful to include international instruments in further study designs.

Despite the diversity of the sample and the rich geographic representations and demographic measures, we cannot exclude that more extreme views on vaccines were not adequately captured or that certain specific subgroups, including rural areas, within the population, were not fully represented. We can infer that certain population groups were more likely to participate in the study than others, such as active Internet users.

Future research tracking changing attitudes toward vaccination will be important as the COVID-19 pandemic and its vaccination campaign continue.

## Data Availability Statement

The original contributions presented in the study are included in the article/Supplementary Material, further inquiries can be directed to the corresponding author.

## Ethics Statement

The participants provided their written informed consent to participate in this study. The Independent Ethical Committee at the V. M. Bekhterev National Medical Research Center approves the study for Psychiatry and Neurology (EK-I-31/21 from 25.02.2021).

## Author Contributions

AV and TK designed the study and wrote the first draft of the manuscript. AV, TK, DR, IM, SB, KN, and EC performed the study. AY undertook the imaging data analysis. EC, AV, and DR revised the manuscript. All authors contributed to the article and approved the submitted version.

## Conflict of Interest

The authors declare that the research was conducted in the absence of any commercial or financial relationships that could be construed as a potential conflict of interest.

## Publisher’s Note

All claims expressed in this article are solely those of the authors and do not necessarily represent those of their affiliated organizations, or those of the publisher, the editors and the reviewers. Any product that may be evaluated in this article, or claim that may be made by its manufacturer, is not guaranteed or endorsed by the publisher.

## Abbreviations

COVID-19: coronavirus disease 2019
WHO: World Health Organization.
