# Supplementary material for: Concerns and Challenges Related to Sputnik V Vaccination Against the Novel COVID-19 Infection in the Russian Federation: The Role of Mental Health, and Personal and Social Issues as Targets for Future Psychosocial Interventions
Source: Front Psychiatry. 2022 Jun 14;13:835323. doi: 10.3389/fpsyt.2022.835323 (PMC9237238; doi:10.3389/fpsyt.2022.835323)
Supplement: Supplementary file 1 [file Table_1.docx]

Supplementary table 1: Association between COVID-19 vaccination attitudes and respondents’ readiness to recommend an immunization to their relatives

| The attitude of the population to vaccination against COVID-19 (Q2_015) | | | Readiness to recommend immunization to the relatives (Q2_020) | | | | Total  sample n (%) |
| --- | --- | --- | --- | --- | --- | --- | --- |
|  |  |  | Intend to actively dissuade | Not ready to recomm end | Not yet decided on a  decision | Recommend a  vaccine |  |
|  | Vaccination is unnecessary | Sample (n) | 74 | 319 | 56 | 43 | 492 |
|  |  | Q2_015 | 15.0% | 64.8% | 11.4% | 8.7% | 100.0% |
|  |  | Q2_020 | 25.3% | 16.1% | 4.1% | 3.2% | 9.9% |
|  |  | Total sample % | 1.5% | 6.4% | 1.1% | 0.9% | 9.9% |
|  | Vaccination is useful | Sample (n) | 1 | 152 | 370 | 1180 | 1703 |
|  |  | Q2_015 | 0.1% | 8.9% | 21.7% | 69.3% | 100.0% |
|  |  | Q2_020 | 0.3% | 7.7% | 27.2% | 88.1% | 34.2% |
|  |  | Total sample (%) | 0.0% | 3.1% | 7.4% | 23.7% | 34.2% |
|  | Vaccination is dangerous | Sample (n) | 159 | 385 | 61 | 4 | 609 |
|  |  | Q2_015 | 26.1% | 63.2% | 10.0% | 0.7% | 100.0% |
|  |  | Q2_020 | 54.3% | 19.4% | 4.5% | 0.3% | 12.2% |
|  |  | Total sample (%) | 3.2% | 7.7% | 1.2% | 0.1% | 12.2% |
|  | Doubts about the effectiven ess | Sample (n) | 47 | 858 | 574 | 71 | 1550 |
|  |  | Q2_015 | 3.0% | 55.4% | 37.0% | 4.6% | 100.0% |
|  |  | Q2_020 | 16.0% | 43.2% | 42.3% | 5.3% | 31.1% |
|  |  | Total sample (%) | 0.9% | 17.2% | 11.5% | 1.4% | 31.1% |
|  | Indifferent attitude | Sample (n) | 4 | 173 | 170 | 20 | 367 |
|  |  | Q2_015 | 1.1% | 47.1% | 46.3% | 5.4% | 100.0% |
|  |  | Q2_020 | 1.4% | 8.7% | 12.5% | 1.5% | 7.4% |
|  |  | Total sample (%) | 0.1% | 3.5% | 3.4% | 0.4% | 7.4% |
|  | Others | Sample (n) | 8 | 99 | 127 | 22 | 256 |
|  |  | Q2_015 | 3.1% | 38.7% | 49.6% | 8.6% | 100.0% |
|  |  | Q2_020 | 2.7% | 5.0% | 9.4% | 1.6% | 5.1% |
|  |  | Total sample (%) | 0.2% | 2.0% | 2.6% | 0.4% | 5.1% |
| Total sample | | Sample (n) | 249 | 293 | 1986 | 1358 | 1340 |
|  |  | Q2_015 | 6.0% | 5.9% | 39.9% | 27.3% | 26.9% |
|  |  | Q2_020 | 100.0% | 100.0  % | 100.0  % | 100.0% | 100.0% |
|  |  | Total sample (%) | 6.0% | 5.9% | 39.9% | 27.3% | 26.9% |
