# Supplementary material for: Concerns and Challenges Related to Sputnik V Vaccination Against the Novel COVID-19 Infection in the Russian Federation: The Role of Mental Health, and Personal and Social Issues as Targets for Future Psychosocial Interventions
Source: Front Psychiatry. 2022 Jun 14;13:835323. doi: 10.3389/fpsyt.2022.835323 (PMC9237238; doi:10.3389/fpsyt.2022.835323)
Supplement: Supplementary file 2 [file Table_2.docx]

Supplementary table 2: Association between the factors of attitudes towards COVID-19 vaccination and fears of complications caused by the vaccination

| Attitudes towards COVID-19 vaccination (Q2_015) | | | «Are you afraid of complications from vaccination? » Q2_024 | | | | | Total sample n (%) |
| --- | --- | --- | --- | --- | --- | --- | --- | --- |
|  |  |  | «No,  I'm not afraid» (0) | «A little apprehe nsive» (1) | «Moderat ely apprehens ive»  (2) | «I am afraid» (3) | «I am very  afraid» (4) |  |
|  | Vaccination is unnecessary | Sample (n) | 117 | 73 | 80 | 94 | 128 | 492 |
|  |  | Q2_015 | 23.8% | 14.8% | 16.3% | 19.1% | 26.0% | 100.0  % |
|  |  | Q2_024 | 12.4% | 5.4% | 6.9% | 11.7% | 17.7% | 9.9% |
|  |  | Total sample (%) | 2.4% | 1.5% | 1.6% | 1.9% | 2.6% | 9.9% |
|  | Vaccination is useful | Sample (n) | 538 | 722 | 345 | 77 | 21 | 1,703 |
|  |  | Q2_015 | 31.6% | 42.4% | 20.3% | 4.5% | 1.2% | 100.0  % |
|  |  | Q2_024 | 56.9% | 53.8% | 29.7% | 9.6% | 2.9% | 34.2% |
|  |  | Total sample (%) | 10.8% | 14.5% | 6.9% | 1.5% | 0.4% | 34.2% |
|  | Vaccination is dangerous | Sample (n) | 28 | 36 | 80 | 171 | 294 | 609 |
|  |  | Q2_015 | 4.6% | 5.9% | 13.1% | 28.1% | 48.3% | 100.0  % |
|  |  | Q2_024 | 3.0% | 2.7% | 6.9% | 21.3% | 40.6% | 12.2% |
|  |  | Total sample  (%) | 0.6% | 0.7% | 1.6% | 3.4% | 5.9% | 12.2% |
|  | Doubts about the effectiveness | Sample (n) | 132 | 321 | 502 | 375 | 220 | 1,550 |
|  |  | Q2_015 | 8.5% | 20.7% | 32.4% | 24.2% | 14.2% | 100.0  % |
|  |  | Q2_024 | 14.0% | 23.9% | 43.2% | 46.8% | 30.3% | 31.1% |
|  |  | Total sample (%) | 2.7% | 6.4% | 10.1% | 7.5% | 4.4% | 31.1% |
|  | Indifferent attitude | Sample (n) | 111 | 128 | 72 | 28 | 28 | 367 |
|  |  | Q2_015 | 30.2% | 34.9% | 19.6% | 7.6% | 7.6% | 100.0  % |
|  |  | Q2_024 | 11.7% | 9.5% | 6.2% | 3.5% | 3.9% | 7.4% |
|  |  | Total sample (%) | 2.2% | 2.6% | 1.4% | 0.6% | 0.6% | 7.4% |
|  | Others | Sample (n) | 20 | 62 | 84 | 56 | 34 | 256 |
|  |  | Q2_015 | 7.8% | 24.2% | 32.8% | 21.9% | 13.3% | 100.0  % |
|  |  | Q2_024 | 2.1% | 4.6% | 7.2% | 7.0% | 4.7% | 5.1% |
|  |  | Total sample  (%) | 0.4% | 1.2% | 1.7% | 1.1% | 0.7% | 5.1% |
| Total sample n (%) | | Sample (n) | 946 | 1,342 | 1,163 | 801 | 725 | 4,977 |
|  |  | Q2_015 | 19.0% | 27.0% | 23.4% | 16.1% | 14.6% | 100.0  % |
|  |  | Q2_024 | 100.0  % | 100.0% | 100.0% | 100.0% | 100.0% | 100.0  % |
|  |  | Total sample  (%) | 19.0% | 27.0% | 23.4% | 16.1% | 14.6% | 100.0  % |
