# Supplementary material for: Concerns and Challenges Related to Sputnik V Vaccination Against the Novel COVID-19 Infection in the Russian Federation: The Role of Mental Health, and Personal and Social Issues as Targets for Future Psychosocial Interventions
Source: Front Psychiatry. 2022 Jun 14;13:835323. doi: 10.3389/fpsyt.2022.835323 (PMC9237238; doi:10.3389/fpsyt.2022.835323)
Supplement: Supplementary file 3 [file Table_3.docx]

Supplementary table 3: Association between vaccination attitudes and fears of contamination with COVID-19

| The attitude of the population to vaccination against COVID-19 (Q2_015) | | | | COVID 19 contamination fear (Q2_027) | | | | | Total sample  n. % |
| --- | --- | --- | --- | --- | --- | --- | --- | --- | --- |
|  |  |  |  | Do not have fears | Slightly fear | Moderately | Intense | Very strong |  |
|  | Vaccination is unnecessary | Sample (n) | | 322 | 108 | 42 | 12 | 8 | 492 |
|  |  |  | Q2_015 | 65.4% | 22.0% | 8.5% | 2.4% | 1.6% | 100.0% |
|  |  |  | Q2_027 | 16.5% | 7.4% | 3.5% | 4.8% | 7.5% | 9.9% |
|  |  |  | Total sample (%) | 6.5% | 2.2% | 0.8% | 0.2% | 0.2% | 9.9% |
|  | Vaccination is useful | Sample (n) | | 450 | 523 | 547 | 132 | 51 | 1,703 |
|  |  |  | Q2_015 | 26.4% | 30.7% | 32.1% | 7.8% | 3.0% | 100.0% |
|  |  |  | Q2_027 | 23.0% | 36.1% | 45.0% | 52.6% | 47.7% | 34.2% |
|  |  |  | Total sample (%) | 9.0% | 10.5% | 11.0% | 2.7% | 1.0% | 34.2% |
|  | Vaccination is dangerous | Sample (n) | | 297 | 151 | 122 | 24 | 15 | 609 |
|  |  |  | Q2_015 | 48.8% | 24.8% | 20.0% | 3.9% | 2.5% | 100.0% |
|  |  |  | Q2_027 | 15.2% | 10.4% | 10.0% | 9.6% | 14.0% | 12.2% |
|  |  |  | Total sample (%) | 6.0% | 3.0% | 2.5% | 0.5% | 0.3% | 12.2% |
|  | Doubts about the effectivene ss | Sample (n) | | 593 | 492 | 373 | 64 | 28 | 1,550 |
|  |  |  | Q2_015 | 38.3% | 31.7% | 24.1% | 4.1% | 1.8% | 100.0% |
|  |  |  | Q2_027 | 30.4% | 33.9% | 30.7% | 25.5% | 26.2% | 31.1% |
|  |  |  | Total sample (%) | 11.9% | 9.9% | 7.5% | 1.3% | 0.6% | 31.1% |
|  | Indifferent attitude | Sample (n) | | 208 | 97 | 54 | 5 | 3 | 367 |
|  |  |  | Q2_015 | 56.7% | 26.4% | 14.7% | 1.4% | 0.8% | 100.0% |
|  |  |  | Q2_027 | 10.7% | 6.7% | 4.4% | 2.0% | 2.8% | 7.4% |
|  |  |  | Total sample (%) | 4.2% | 1.9% | 1.1% | 0.1% | 0.1% | 7.4% |
|  | Others | Sample (n) | | 83 | 79 | 78 | 14 | 2 | 256 |
|  |  |  | Q2_015 | 32.4% | 30.9% | 30.5% | 5.5% | 0.8% | 100.0% |
|  |  |  | Q2_027 | 4.2% | 5.4% | 6.4% | 5.6% | 1.9% | 5.1% |
|  |  |  | Total sample (%) | 1.7% | 1.6% | 1.6% | 0.3% | 0.0% | 5.1% |
| Total sample | | | Sample (n) | 1,515 | 1,953 | 1,450 | 1,216 | 251 | 107 |
|  |  |  | Q2_015 | 36.3% | 39.2% | 29.1% | 24.4% | 5.0% | 2.1% |
|  |  |  | Q2_027 | 100.0  % | 100.0% | 100.0% | 100.0  % | 100.0  % | 100.0% |
|  |  |  | Total sample (%) | 36.3% | 39.2% | 29.1% | 24.4% | 5.0% | 2.1% |
