# Supplementary material for: Concerns and Challenges Related to Sputnik V Vaccination Against the Novel COVID-19 Infection in the Russian Federation: The Role of Mental Health, and Personal and Social Issues as Targets for Future Psychosocial Interventions
Source: Front Psychiatry. 2022 Jun 14;13:835323. doi: 10.3389/fpsyt.2022.835323 (PMC9237238; doi:10.3389/fpsyt.2022.835323)
Supplement: Supplementary file 4 [file Table_4.docx]

Supplementary table 4: Selected variables used in the discriminant model examining the respondents’ attitudes towards vaccination

| № | Variables group description | Variables included in the group |
| --- | --- | --- |
| 1 | Age-gender, social respondents characteristics | - Gender (Q1_001) - m/w - Age according WHO classification (Q1_S_002) - Education (Q1_003) - Education type, if higher (Q1_004) - Social status (Q1_005) - Type of residence (Q1_008) - If the respondent is a healthcare worker (Q1_009а) – yes/no - Marital status (Q1_010) - Living conditions (Q1_011) |
| 2 | COVID-19 personal experience | - Was the respondent contracted by COVID-19 and how severe (Q2_013) - Was someone in the respondent’s close environment (relatives, friends) who sufferd from COVID-19 severe   form or died (Q2_014а) |
| 3 | Presence of chronic diseases, mental health problems, fears associated with COVID-19 | - Presence of chronic diseases (Q2_025) - Presence of mental health problems (Q2_026) - Coronaphobia (Q2_027) - Worries about family members (contraction or death of COVID-19) (Q2_028) - Anxiety related to COVID-19 (Q2_029) - Tanatophobia related to COVID-19 (Q2_030) |
| 4 | Presence/ Absence of wide- spread vaccination beliefs.  **Comment:** the resent study has used The Vaccination Attitudes Examination (VAX) Scale for the evaluation of the vaccination beliefs. However, in the implementation of this model the same information  can be got from the social networks. | The Vaccination Attitudes Examination (VAX) Scale:   - Mistrust of vaccine benefit (Q3_S_1) - Worries about unforeseen future effects (Q3_S_2) - Concerns about commercial profiteering (Q3_S_3) - Preference for natural immunity (Q3_S_4) |
| 5 | General psychological well- being or distress state | General health questionnaire 12 (GHQ-12):   - General psychological well-being or distress state (Q4_S) |
| 6 | Attitude to one’s health | Attitude to one’s health questionnaire scales (R. A. Berezovskaya)   - Cognitive scale (Q5_S1) - Emotional scale (Q5_S2) - Behavior scale(Q5_S3) - Values-motivation scale (Q5_S4) |
