# Supplementary material for: Concerns and Challenges Related to Sputnik V Vaccination Against the Novel COVID-19 Infection in the Russian Federation: The Role of Mental Health, and Personal and Social Issues as Targets for Future Psychosocial Interventions
Source: Front Psychiatry. 2022 Jun 14;13:835323. doi: 10.3389/fpsyt.2022.835323 (PMC9237238; doi:10.3389/fpsyt.2022.835323)
Supplement: Supplementary file 5 [file Table_5.docx]

Supplementary table 5: Canonical discriminant function coefficients (unstandardized) for selected variables used in the model examining the respondents’ attitudes towards vaccination

| Variables used in the model (see Table 8) | Canonical discriminant function | | | | |
| --- | --- | --- | --- | --- | --- |
|  | 1 | 2 | 3 | 4 | 5 |
| Q1_001 | ,163 | -,357 | -,401 | -,247 | ,725 |
| Q1_S_002 | ,209 | ,160 | -,333 | ,652 | ,059 |
| Q1_003 | ,121 | ,372 | -,351 | ,111 | ,179 |
| Q1_004 | ,007 | -,067 | ,007 | -,049 | ,265 |
| Q1_005 | ,057 | ,072 | -,072 | -,203 | -,124 |
| Q1_008 | ,024 | ,076 | ,085 | ,014 | -,213 |
| Q1_009a | ,099 | ,325 | ,081 | 1,222 | ,198 |
| Q1_010 | -,011 | ,063 | ,235 | ,060 | ,025 |
| Q1_011 | ,000 | ,074 | -,170 | -,168 | ,227 |
| Q2_013 | ,010 | ,120 | -,007 | ,021 | ,038 |
| Q2_014a | ,158 | ,488 | ,148 | -,046 | -,319 |
| Q2_025 | -,006 | ,403 | ,188 | -,116 | ,356 |
| Q2_026 | ,003 | ,080 | -,087 | -,226 | ,252 |
| Q2_027 | ,061 | ,314 | -,128 | -,011 | ,242 |
| Q2_028 | ,008 | ,008 | ,419 | ,132 | ,192 |
| Q2_029 | ,128 | ,136 | -,247 | ,122 | ,008 |
| Q2_030 | -,095 | -,040 | -,140 | -,081 | -,001 |
| Q3_S_1 | ,227 | -,040 | ,103 | -,131 | ,114 |
| Q3_S_2 | -,060 | ,150 | ,112 | -,173 | -,002 |
| Q3_S_3 | -,091 | ,012 | ,054 | ,023 | ,237 |
| Q3_S_4 | -,026 | -,034 | -,081 | ,007 | -,098 |
| Q4_S | -,010 | ,007 | ,058 | ,022 | -,059 |
| Q5_S1 | -,002 | ,006 | ,018 | ,022 | -,020 |
| Q5_S2 | -,002 | -,007 | -,021 | ,022 | ,008 |
| Q5_S3 | ,000 | -,001 | -,014 | -,008 | ,014 |
| Q5_S4 | ,002 | ,002 | -,007 | ,006 | ,002 |
| (Constant) | -1,398 | -4,162 | -,751 | -,997 | -3,586 |
