# Supplementary material for: Concerns and Challenges Related to Sputnik V Vaccination Against the Novel COVID-19 Infection in the Russian Federation: The Role of Mental Health, and Personal and Social Issues as Targets for Future Psychosocial Interventions
Source: Front Psychiatry. 2022 Jun 14;13:835323. doi: 10.3389/fpsyt.2022.835323 (PMC9237238; doi:10.3389/fpsyt.2022.835323)
Supplement: Supplementary file 6 [file Table_6.docx]

Supplementary table 6: Group centroids of discriminant functions 1-5 used in the model examining the respondents’ attitudes towards vaccination

| Group of respondents (see Table 7) | Group means of unstandardized canonical discriminant functions | | | | |
| --- | --- | --- | --- | --- | --- |
|  | 1 | 2 | 3 | 4 | 5 |
| 1 | -1,251 | -,456 | -,333 | ,068 | -,047 |
| 2 | 1,253 | -,009 | -,058 | -7,790E-6 | ,033 |
| 3 | -1,449 | ,325 | -,045 | -,130 | ,117 |
| 4 | -,391 | ,143 | ,118 | ,118 | -,035 |
| 5 | -,346 | -,749 | ,325 | -,155 | ,022 |
| 6 | ,248 | ,336 | -,048 | -,321 | -,225 |
