# Supplementary material for: Concerns and Challenges Related to Sputnik V Vaccination Against the Novel COVID-19 Infection in the Russian Federation: The Role of Mental Health, and Personal and Social Issues as Targets for Future Psychosocial Interventions
Source: Front Psychiatry. 2022 Jun 14;13:835323. doi: 10.3389/fpsyt.2022.835323 (PMC9237238; doi:10.3389/fpsyt.2022.835323)
Supplement: Supplementary file 7 [file Table_7.docx]

Supplementary table 7: Results of assessing the quality of the developed classification model

| Original group of  respondents | | Predicted group membership | | | | | | Total |
| --- | --- | --- | --- | --- | --- | --- | --- | --- |
|  |  | 1 | 2 | 3 | 4 | 5 | 6 |  |
| Count | 1 | 188 | 45 | 129 | 26 | 66 | 21 | 475 |
|  | 2 | 21 | 1180 | 10 | 65 | 143 | 270 | 1689 |
|  | 3 | 91 | 19 | 301 | 60 | 62 | 63 | 596 |
|  | 4 | 187 | 167 | 280 | 304 | 279 | 312 | 1529 |
|  | 5 | 48 | 33 | 32 | 27 | 166 | 48 | 354 |
|  | 6 | 11 | 68 | 29 | 24 | 19 | 100 | 251 |
| % | 1 | 39,6 | 9,5 | 27,2 | 5,5 | 13,9 | 4,4 | 100,0 |
|  | 2 | 1,2 | 69,9 | ,6 | 3,8 | 8,5 | 16,0 | 100,0 |
|  | 3 | 15,3 | 3,2 | 50,5 | 10,1 | 10,4 | 10,6 | 100,0 |
|  | 4 | 12,2 | 10,9 | 18,3 | 19,9 | 18,2 | 20,4 | 100,0 |
|  | 5 | 13,6 | 9,3 | 9,0 | 7,6 | 46,9 | 13,6 | 100,0 |
|  | 6 | 4,4 | 27,1 | 11,6 | 9,6 | 7,6 | 39,8 | 100,0 |
